# 204 Physical activity insecurity in children and young people at risk of marginalisation: navigating an equitable and safe research experience using co-production principles

**DOI:** 10.1093/eurpub/ckae114.097

**Published:** 2024-09-26

**Authors:** Caroline Dodd-Reynolds, Scarlet Hall, Mary Crowder, Victoria Goodyear, Naomi Griffin, Hannah Fairbrother, Stacey Pope, Steph Scott

**Affiliations:** Durham University, Durham, UK; Durham University, Durham, UK; University of Sheffield, Sheffield, UK; University of Birmingham, Birmingham, UK; Newcastle University, Newcastle upon Tyne, UK; University of Sheffield, Sheffield, UK; Durham University, Durham, UK; Newcastle University, Newcastle upon Tyne, UK

## Abstract

**Purpose:**

We recently proposed a new conceptualisation for physical activity (PA) insecurity as ‘a limited or restricted ability to be active, reinforced by worries and experiences of feeling uncomfortable, emotionally or physically unsafe’ (Dodd-Reynolds et al., 2024). We suggested that PA insecurity can be related to disadvantage, lack of inclusivity and may be particularly heightened for LGBTQ+ young people.

The Joyful and Safe Physical Activity “JASPA project” aims to develop recommendations to make physical activity more inclusive and safe for LGBTQ+ young people living with disadvantage, via a series of participatory creative visual methods workshops. At the heart of the project lies a desire to create safe spaces for young people to share their experiences and ideas without exacerbating existing worries and fears. Here we present JASPA as a case study to reflect on how principles of co-production can support physical activity research with young people at risk of marginalisation.

**Methods:**

We adopted NIHR (2021) principles on co-producing a research project: sharing of power, including all perspectives and skills, respecting and valuing knowledge of those working together on the research, reciprocity, and building and maintaining relationships. These principles were front and centre of researcher meetings, building trust with organisations and young people, development of creative visual methods workshops, and analyses.

**Results:**

Challenges and opportunities have been numerous and insightful, including institutional power and parental consent; questioning bounded roles and balance of power across researchers and facilitators, as well as participants/co-researchers; iterative participant-researcher-led fieldwork practices to raise difficult conversations; engaging in continual and necessary reflexivity.

**Conclusions:**

In our experience, co-production can be ‘messy’, time-consuming, costly and demanding for all involved, but also offers real and desirable opportunities for innovation in PA research. Using the JASPA project as a case study, we suggest a range of mechanisms to support this process including building in time to develop relationships and build trust, devising working principles for researchers, and participants/co-researchers, flexible methodological boundaries, and debrief activities.

**Funding Source:**

This study/project is funded/supported by the National Institute for Health and Care Research (NIHR) School for Public Health Research (SPHR) (Grant Reference Number NIHR 204000).

